# How to Dax? Preschool Children’s Prosocial Behavior, But Not Their Social Norm Enforcement Relates to Their Peer Status

**DOI:** 10.3389/fpsyg.2017.01779

**Published:** 2017-11-03

**Authors:** Markus Paulus

**Affiliations:** Department of Psychology, Ludwig-Maximilians-Universität München, Munich, Germany

**Keywords:** prosocial behavior, preschool children, social status, rule following, normativity

## Abstract

The current study examined correlates of preschool children’s (*n* = 82) peer status. In particular, we assessed children’s prosocial behavior, social problem behavior, norm enforcement, language abilities, and temperament. Children’s prosocial behavior, pragmatic language abilities, and gender correlated with peer status. A regression analysis revealed that prosocial behavior and gender were independent predictors. There was some evidence for a mediation effect: The link between pragmatic language and peer status was mediated by prosocial behavior. Children’s norm enforcement was not related to peer status, neither was it related to any other factor such as temperament or language. Overall, the study supports approaches claiming that prosocial behavior plays a role in children’s social functioning and are in line with social-interactionist accounts to social and social-cognitive development.

## Introduction

Children’s status among their peers plays an important role in their social development. Children who are more popular among their peers are more likely to show adaptive social behavior and are less likely to develop behavioral problems (Newcomb et al., 1993). Moreover, children’s positive social relationships with their peers at one stage predict their social adjustment when transitioning to another social institution (Ladd and Price, 1987). Consequently, developmental psychology has been interested in the factors that relate to children’s peer status (e.g., Russell and Finnie, 1990; Arsenio et al., 2000; Slaughter et al., 2015; Daniel et al., 2016).

Research has provided evidence that the propensity to engage in prosocial behavior (that is, behavior benefitting another person without receiving an immediate pay-off; Paulus, 2014) is positively related to children’s peer status (e.g., Denham et al., 1990; Warden and Mackinnon, 2003; Caputi et al., 2012), whereas social problem behavior (such as aggression) is negatively linked to peer status (e.g., Slaughter et al., 2002; Keane and Calkins, 2004). Moreover, several studies have suggested relations between children’s temperamental characteristics (that is, individual differences between persons in reactivity, emotionality, and behavioral styles) and their peer status (Eisenberg et al., 1993, 1995; Szewczyk-Sokolowski et al., 2005; Dougherty, 2006; Stright et al., 2008). For example, Eisenberg et al. (1993) found that negative emotionality is related to lower social peer status. Similarly, Szewczyk-Sokolowski et al. (2005) reported that a composite score of difficult temperamental characteristics related negatively to positive peer nominations and positively to negative peer nominations in a peer acceptance measure. In addition, high levels of regulation have been related to adaptive social functioning (Eisenberg et al., 1995). Furthermore, it has been found that age (within a group) relates positively to peer status and that boys are less well accepted than girls (Lemerise, 1997).

From a cognitive point of view, language skills have been related to social competence and peer status (e.g., Howes, 1988; Coie et al., 1990; Rice, 1993; Gertner et al., 1994; Menting et al., 2011). Some studies even reported that an effect of theory-of-mind knowledge on peer status disappears once language is controlled for (Watson et al., 1999), suggesting that children’s language abilities could be an underlying factor driving potential relations between their peer status and other social-cognitive skills. Taken together, current research has identified a number of temperamental, social, and cognitive factors that relate to children’s peer status.

Interestingly, recent research pointed to another phenomenon that is relevant for children’s behavior toward peers. It has been demonstrated that young preschool children show an understanding of norms and enforce social norms toward others (e.g., Rakoczy et al., 2008; Casler et al., 2009; Schmidt et al., 2016; Wörle and Paulus, 2017). For example, Rakoczy et al. (2008) presented 2- and 3-year-old children with novel games that – as all games (cf. Wittgenstein, 1953) – consisted of norms how to play it. Children were familiarized with the rules by an agent and, subsequently, observed another agent performing an incorrect action. Children of both age groups (but more clearly the 3-year-olds) displayed protest behavior against the agent who violated the norm. This work and related studies (for review see Rakoczy and Schmidt, 2013) provide evidence for a normative stance in preschool children.

These findings are interesting as a number of theoretical approaches have suggested that norms are a unique aspect of human life (e.g., Brandom, 1994) and are, from a psychological perspective, central for group cooperation and maintenance as they allow for coordination and cooperation (Boyd and Richerson, 2009; Tomasello, 2009). Moreover, normative understanding reflects that children learn how things ought to be done in a community (Rakoczy and Schmidt, 2013) and become thus able to ensure functioning and enduring cooperation in a group. It has thus been argued that an appreciation of norms is related to becoming a member of a group or possessing group-mindedness (Schmidt and Tomasello, 2012). If the appreciation of social norms plays a functional role for children’s integration into groups and cooperative behavior, one could hypothesize that a grasp of normativity and an enforcement of norms should be related to their peer status in a group.

However, current evidence is inconclusive with respect to this central theoretical claim. There is one study that speaks directly to this debate. Hawley and Geldhof (2012) assessed, inter alia, teacher ratings of social dominance (i.e., power over resources), prosocial strategies, internalized conscience, sociomoral behaviors (e.g., comforting other children, offering reparations after having hurt someone), and selective moral engagement (i.e., a measure including children’s enforcement of norms in their interactions with peers) in preschool children. In addition, children participated in a sociometric nomination procedure to assess their peer popularity and they were interviewed to assess their moral cognitive development. Most interesting, peer popularity was positively related to, inter alia, prosocial strategies, sociomoral behavior (e.g., empathy), moral cognition, and internalized conscience, but not to selective moral engagement, that is, to the measure assessing their norm enforcement. In contrast, a regression analysis revealed that children’s social dominance was positively predicted by their selective moral engagement and negatively related to internalized conscience. This pattern of results might suggest that social norm enforcement could rather play a self-serving role in preschool children, whereas prosocial behavior and moral cognition relate to peer status. However, in this study social norm enforcement behaviors were only one aspect of a broader factor relating to selective moral engagement, yielding thus only preliminary evidence. A study that more directly assesses children’s social norm enforcement would therefore fill a crucial gap in the literature.

The current study was thus designed to explore the question whether or not children’s social norm enforcement is an independent predictor to their peer status next to the already established factors.

### The Current Study

To this end, we examined preschool children’s peer status – here operationally defined by the extent to which other children like to play with a particular child – by means of an established procedure by Asher et al. (1979). Children’s prosocial behavior was assessed by means of the Strength and Difficulties Questionnaire (SDQ; Goodman, 1997). We assessed children’s social norm enforcement by adopting the setup by Rakoczy et al. (2008). Furthermore, to assess whether children’s norm enforcement is a unique predictor of their peer status, we additionally included measures of child temperament, general social problem behavior, and language abilities. An inclusion of these aspects was warranted as children’s likelihood to engage in social norm enforcement could be related to temperamental features (e.g., shy children not engaging in norm enforcement; Rakoczy et al., 2008), general aspects of social behavior (e.g., children with social problem behavior generally being less likely to enforce social norms), or language abilities (as norm enforcement is largely assessed by analyzing children’s verbal utterances). If we were to find relations between children’s social norm enforcement and their peer status, it would be important to clarify whether or not this relation would be independent of the other factors mentioned above. Consequently, we performed a linear regression analysis including these different predictors.

Additionally, assessing these variables would also help us to shed further light on the variance found in children’s propensity to engage in social norm enforcement. It would provide a direct test of the claim that temperamental factors such as shyness might explain individual differences in norm enforcement (Rakoczy et al., 2008). Likewise, as outlined above, language abilities or general social behavioral tendencies could also explain individual differences. To this end, a second aim of the current study was the exploration of potential correlates of children’s social norm enforcement.

## Materials and Methods

### Participants

The final sample consisted of 82 kindergarten children with a mean age of 5.2 years (age range: 3.7–6.8 years; 38 male). Age did not differ between girls (*M* = 5.1, *SD* = 0.9) and boys (*M* = 5.4, *SD* = 1.0), *t*(80) = 1.47, *p* = 0.147. One additional child was tested, but excluded due to an experimenter error. The participants attended 1 of 3 different day care centers in a larger German city (with 21, 27, and 34 children coming from each kindergarten, respectively). Children were native German speakers. The study followed the ethical principals outlined by the Helsinki’s 1964 declaration. Informed consent was given by the children’s caregivers.

### Procedure and Materials

Participants were tested individually in a quiet room. Experimental sessions were videotaped. The experimenter and the child were sitting at a table facing each other. Children were first presented with the peer status assessment and thereafter with the norm enforcement task. The tasks will be described in greater detail below.

#### Questionnaires

Kindergarten teachers filled out three questionnaires. The German version of the SDQ (Goodman, 1997) was applied to assess two aspects of social behavior: prosocial and problematic behavior (Klasen et al., 2003). The SDQ consists of the five subscales *prosocial behavior*, *peer problems*, *emotional symptoms*, *conduct problems*, and *hyperactivity* (Goodman, 1997). The latter four subscales are combined to yield a measure of problem behaviors. Child temperament was assessed by the German translation of Buss and Plomin (1984) EAS Temperament Survey (Angleitner et al., 1991, unpublished). The EAS assesses temperament on four dimensions: emotionality, activity, sociability, and shyness. Each dimension consists of five items that are answered from a 1- to 5-point rating scale. Child language abilities were assessed by means of the *Kompik Inventory* (Kompetenzen und Interessen von Kindern in Kindertageseinrichtungen), a measure of child competencies across several domains (Mayr et al., 2011; Mayr, 2012). For the purpose of the present study, two subscales of the language domain were chosen: the “grammar” subscale that assesses children’s performance with respect to correct grammatics and the “speaking and understanding” subscale that assesses children’s ability to correctly understand and engage in verbal interactions (henceforth labeled pragmatic language). Each scale includes five items that are rated on a 5-point scale. Scoring of all questionnaires was based on the guidelines of the respective tool.

#### Peer Status

After a short warming up with the experimenter, children participated in a rating of the other children of the same kindergarten that also participated in the current study. Adapting a method by Asher et al. (1979), children were asked to rate how much they like to play with the respective other child on a 4-point scale. To support children’s judgments, a smiley scale was presented. It consisted of four smileys with the leftmost smiley depicting a very sad face and the rightmost smiley depicting a very happy face. The experimenter presented participants with the names of the other children (one after the other) and participants were asked to point to the respective smiley depending on whether they did not like to play with the respective child at all (1 point, leftmost smiley) to very much (4 points, rightmost smiley).

#### Norm Enforcement and Protest

The norm enforcement measure followed the experimental conditions of two games (daxing; baffing) described by Rakoczy et al. (2008). The details of these tasks will be described further below.

Before the test trials, children were presented with three warm-up trials. In the first and the third of the trials one of the puppets made instrumental mistakes. The warm-up trials were: (1) One puppet made a drawing with a sharp pencil and the second puppet tried to make a drawing with a broken pencil. (2) The first puppet opened a box with a complex mechanism. The second puppet correctly imitated the action. (3) One puppet put a toy car into a long and small tube, and pushed it to the other hand by means of a stick. The second puppet tried to push the car through the tube with her hand. The warm-up trials familiarized children with the situation, the puppets, and the possibility that they can intervene. If the participant did not intervene within a few seconds (in the first and third trial), the experimenter asked whether the puppet was making any mistake (“Is it correct what < name of the puppet > is doing?”), prompted the participant to correct her (“Can you help < name of the puppet > to do it correctly?”), and, eventually, asked the child how the action works (“Do you know, how it works?”).

The test trials presented children with two novel activities, daxing (in German: “daxen”) and baffing (in German: “baffen”). Following Rakoczy et al. (2008), materials for daxing included a Styrofoam board (with a gutter), a wooden block, a wooden staff, and another small piece of wood. By means of a Velcro tape, the last two items could be put together to form a small racket. The following game was demonstrated: The staff and the small piece of wood were put together to form a racket. The wooden block were to be placed on the Styrofoam board and it had to be pushed into the gutter by means of the racket. The game was introduced in the following manner: Before engaging into this activity, the protagonist announced to the child that he/she was going to demonstrate something. Then, he/she announced that he/she was to demonstrate how Daxing goes. She assembled the objects as described above and executed the respective action. While doing so, she said: “I am daxing.” After she was done, she confirmed: “I just daxed.” She then engaged in another activity (lifting the board so that the wooden block slid off) and confirmed: “Oh no. This is not how daxing goes.” The sequence of these two actions was then repeated another time. Thereafter, the child had the opportunity to perform the action him-/herself. Subsequently, the other protagonist appeared at the stage, announced that he/she was going to engage in Daxing and executed two times the incorrect action while stating “I am daxing” and “I just daxed.”

Materials for baffing included three wooden blocks (one of them longer than the other two), play dough, and a cylinder-like roll. The three wooden blocks were used to build a goal. A small ball was created with the play dough. By means of the roll, the ball could be kicked into the goal. The procedure of this trial followed the Daxing trial with the exception that the game was called Baffing. The incorrect action consisted of building the goal from two wooden blocks only and rolling the roll through the goal by hand.

### Coding and Data Analysis

#### Peer Status

For each participant, an average score based on his/her ratings given by the other children was calculated.

#### Norm Enforcement and Protest

For each phase of each trial, we coded whether children showed no protest (0), imperative protest (1), or normative protest (2). Imperative protest was defined as verbal or active protest (such as an imperative) without normative comments (e.g., “No, not in that way.”). Normative protest was defined as protest that relied on normative vocabulary (e.g., “That is incorrect. You have to do it like that.”). Twenty-eight cases were coded by a second coder. Kappas were 0.834, 0.678, 0.889, and 0.832, respectively. Each trial got then the highest category code that appeared in the two repetitions as its code. We calculated two overall scores. One score denoted the number of trials (out of two) in which children showed any kind of imperative or normative protest (henceforth: imperative protest score). The other score focused solely on normative protest and denoted the number of trials (out of two) in which children showed normative protest (henceforth: normative protest score).

#### Social Behavior

The four problem behavior scales of the SDQ were summed to yield a general problem behavior score. The prosocial behavior subscale was used as an index of children’s prosocial behavior.

#### Child Temperament

For each scale (emotionality, activity, sociability, and shyness), items were summed to build scale scores.

#### Child Language

For each scale (grammar; speaking and understanding), items were summed to build scale scores. Four children did not contribute data to the speaking and understanding scale five children did not contribute data to the grammar scale.

## Results

### Descriptives

**Table 1** presents descriptives of all measures (please see original data in the **Supplementary Data Sheet 1**). **Table 2** presents a full correlation matrix for all variables (please find **Table 2** below). This first overview indicates relations between children’s peer status and their gender (with girls having a higher status, *M* = 2.59, *SD* = 0.27, *range* 2.00–3.20, than boys, *M* = 2.39, *SD* = 0.33, *range* 1.63–2.93), their problem behavior, their prosocial behavior, and their pragmatic language abilities. Notably, the correlation between normative protest and peer status (95% CI; [-0.115, 0.314]) as well as imperative protest and peer status (95% CI; [-0.239, 0.194]) was not significant.

**Table 1:** Descriptives of the main variables.

	Mean	Range	*SD*
Peer status	2.50	1.63–3.20	0.32
Prosocial behavior (SDQ)	7.62	0–10	2.31
Problem behavior (SDQ)	7.74	0–19	4.67
Shyness (EAS)	12.39	5–24	4.65
Emotionality (EAS)	11.67	5–21	4.14
Sociability (EAS)	17.90	13–23	2.48
Activity (EAS)	17.23	5–25	4.48
Grammar (Kompik)	20.06	12–25	4.25
Pragmatic language (Kompik)	20.96	9–25	3.67
Normative protest	0.63	0–2	0.83
Imperative protest	1.00	0–2	0.96

**Table 2:** Full correlational matrix of all variables.

	1	2	3	4	5	6	7	8	9	10	11	12
2	0.137	–										
3	-0.327^∗∗^	0.162	–									
4	-0.211^+^	-0.235^∗^	0.225^∗^	–								
5	0.333^∗∗^	0.207	-0.220^∗^	-0.388^∗∗^	–							
6	-0.010	-0.296^∗∗^	-0.142	0.058	-0.231^∗^	–						
7	-0.005	0.224^∗^	0.067	-0.254^∗^	0.218^∗^	-0.735^∗∗^	–					
8	0.071	-0.129	0.110	0.525^∗∗^	-0.036	-0.069	-0.136	–				
9	0.042	0.226^∗^	0.226^∗^	0.135	-0.128	-0.574^∗∗^	0.548^∗∗^	0.044	–			
10	0.080	0.528^∗∗^	0.103	-0.354	0.115	-0.262^∗^	0.191	-0.100	0.085	–		
11	0.225^∗^	0.370^∗∗^	-0.011	-0.438^∗∗^	0.262^∗^	-0.580^∗∗^	0.492^∗∗^	-0.223	0.389^∗∗^	0.655^∗∗^	–	
12	0.104	0.140	0.087	-0.041	0.108	-0.133	0.176	-0.152	0.040	0.034	0.091	–
13	0.024	0.140	0.026	0.019	-0.039	-0.075	0.063	-0.097	0.058	0.055	0.056	0.752^∗∗^

*1, Social status; 2, age; 3, gender; 4, problem behavior (SDQ); 5, prosocial behavior (SDQ); 6, shyness (EAS); 7, sociability (EAS); 8, emotionality (EAS); 9, activity (EAS); 10, grammar (Kompik); 11, pragmatic language (Kompik); 12, normative protest; 13, imperative protest. ^∗∗^*p* < 0.01; ^∗^*p* < 0.05; ^+^*p* < 0.10*.

### Regression Analysis

To assess the relative contributions of these factors to explain variance in children’s peer status, we conducted a linear regression analysis using the stepwise method. The stepwise method identifies the strongest and significant predictors while removing those that are non-significant. We used the imperative protest score instead of the normative protest score as pure normative protest happened rarely. Importantly, the results stayed the same irrespective of which protest score we used. Missing values led to pairwise exclusion in order to retain higher statistical power. Notably, the pattern of results remained the same when using listwise exclusion. Moreover, the pattern of results remained the same when adding age as either one additional factor or when including age in a first step and then using stepwise method for the remaining factors in a second step: age was not significant, whereas prosocial behavior and gender remained significant factors. The model with Prosocial behavior and Gender resulted as highly significant and explained 17.9% of the variance in children’s peer status (**Table 3**). Notably, children’s protest behavior was not related to their peer status.

**Table 3:** Hierarchical regression analysis to predict child peer status.

	β	Change in *R*^2^	*F*-value	*R*^2^ sum
Prosocial behaviour	0.28^∗^	11.1%		
Gender	-0.27^∗^	6.8%		
			8.06^∗∗^	
				17.9%

^∗∗^*p < 0.01, two-tailed; ^∗^*p* < 0.05, two-tailed*.

Given that some of the variables (i.e., both language-related measures; shyness, sociability, and activity) were strongly intercorrelated, we wanted to ensure that our results are not artificial effects of collinearity. To this end, we reran the regression analysis and excluded the scales grammar, shyness, and activity. The pattern of the results stayed the same.

It should be noted that pragmatic language abilities were a correlate of children’s peer status, whereas it did not turn out to be an independent predictor in the regression analysis. Yet, pragmatic language abilities were related to prosocial behavior that, in turn, was an independent predictor. This pattern of results is suggestive for a mediation effect according to which the impact of pragmatic language abilities on children’s peer status is mediated by prosocial behavior. To substantiate this point, we conducted an exploratory mediation analysis.

Analyses were conducted using bootstrapping procedures (Preacher and Hayes, 2004) and operationalized in SPSS (Preacher and Hayes, 2008). We used 5,000 bootstrap resamples of the data. Statistical significance with alpha at 0.05 is indicated by the 95% confidence intervals not crossing zero.

**FIGURE 1:**
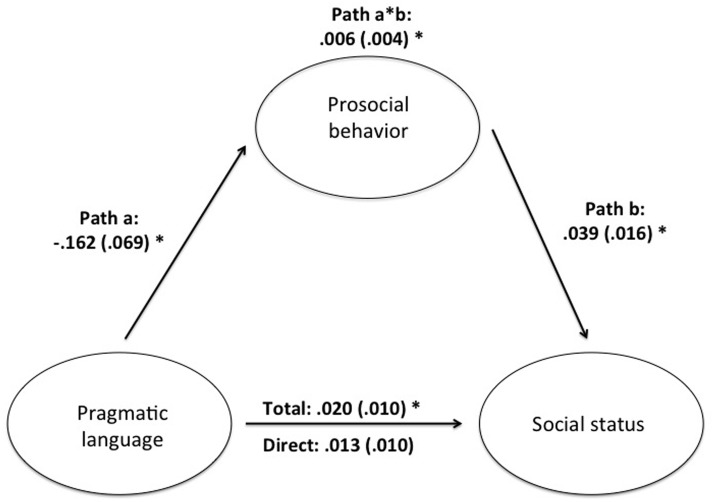
Mediation model. Values are standardized regression coefficients and asterisks indicate significant coefficients (*p* < 0.05).

We found a significant mediation effect of prosocial behavior with respect to the relationship between pragmatic language and peer status (indirect effect = 0.006, *SE* = 0.004, 95% confidence intervals = 0.0003, 0.0180; see **Figure 1**). Mediation was full, meaning that the direct effect of pragmatic language alone did not predict significant portions of the variance observed in peer status (direct effect = 0.013, *SE* = 0.010, 95% confidence intervals = -0.0062, 0.0325).

### Correlates of Children’s Protest Behavior

In an additional analysis, we explored whether children’s propensity to engage in protest behavior was predicted by any of the other variables. For theoretical reasons, potential relations with temperament or language abilities were particularly interesting. Theoretically most interesting, the correlation between normative protest and shyness (95% CI; [-0.340, 0.086]) as well as imperative protest and shyness (95% CI; [-0.287, 0.144]) was not significant. In line with the lack of correlations in the correlation matrix (**Table 1**), regressions on both normative protest and imperative protest yielded no significant predictors. Thus, protest behavior was not related to temperamental factors, children’s age, or their language abilities.

## Discussion

The current study investigated the correlates of preschool children’s peer status. Our results demonstrate that children’s propensity to engage in prosocial behavior and their gender were related to higher peer status with girls being more popular than boys and prosocial children being more popular than less prosocial children. A further correlate was children’s pragmatic language ability. Yet, in a regression analysis it did not turn out as an independent predictor. Interestingly, children’s social norm enforcement did not relate to their peer status. We will discuss the central findings in greater detail in the following paragraphs.

### Which Factors Relate to Preschool Children’s Peer Status?

Our results do support notions that prosocial behavior plays an important role in children’s peer status (e.g., Denham et al., 1990; Caputi et al., 2012). Several routes could explain such a relation. First, children’s empathetic and sympathetic reactions to others might be appreciated by their peers and could lay the basis for more intense contacts and exchange. Second, children’s generosity to share with others could also contain strategic elements such as considerations with whom to share more (e.g., Moore, 2009; Kenward et al., 2015) and from whom to expect reciprocity (e.g., Paulus, 2016) that ultimately lead to reciprocal ties between children that – in turn – could relate to their peer status. Given that prosocial behavior comprises different domains and aspects (e.g., Dunfield, 2014; Paulus, 2014, 2018; Abramson et al., 2017) future research needs to study more carefully how each of these domains might relate to children’s peer status. However, it should be noted that our results are of correlational nature. It could thus well be that a higher peer status in their peer group leads children to be more prosocial toward others or that another characteristic of social development (e.g., attachment) might underlie the development of both aspects (but for evidence for a causal effect of prosocial behavior on peer status see Layous et al., 2012). Further longitudinal research is necessary to single out the exact developmental pathways.

Our results also corroborate findings that girls have higher peer status than boys (Lemerise, 1997). Given that gender itself is not a behavioral category that could explain children’s peer status, we have to consider the psychological processes. One possibility is that girls might show enhanced prosocial behavior (for mixed evidence see Eisenberg and Lennon, 1983), which in turn could result in a higher peer status. Yet, the result of the regression analysis renders this interpretation unlikely as gender remained a significant factor even after accounting for participants’ prosocial behavior. Relatedly, it is possible that girls’ reduced externalizing problem behavior (Eisenberg et al., 2001; Miner and Clarke-Stewart, 2008) could explain this relation as problem behavior has been negatively linked to peer status (Keane and Calkins, 2004) and was – in our sample – related to child gender.

Although it was no independent predictor in the regression analysis, it should be acknowledged that children’s language abilities were correlated with their peer status. More specifically, their pragmatic language abilities but not their grammar were related to their peer status. Given that pragmatic abilities were positively related to prosocial behavior and negatively related to social problem behavior, we tested for a potential mediation effect. This exploratory analysis indeed pointed to an indirect effect of pragmatic language abilities through prosocial behavior on children’s peer status. These findings relate well to social-interactionist proposals that language helps children to successfully navigate the social world, understand and relate to others, and therefore establish relationships (e.g., Carpendale and Lewis, 2004). It is not surprising that pragmatic abilities play a greater role for successful social interactions than correct grammar. Further longitudinal studies are needed to confirm this finding.

Interestingly, we did not find evidence that children’s social norm enforcement was related to their peer status. This finding adds to the recent debate on the nature and function of young children’s norm enforcements (e.g., Tomasello, 2014). More concretely, it has been proposed that children’s norm enforcement indicates a we-intentionality and group-mindedness (Schmidt and Tomasello, 2012; Tomasello, 2014). We would have therefore expected that their inclination to show norm enforcement would be reflected in their status in the group. Yet, our results do not support this consideration. This is in line with a previous study by Hawley and Geldhof (2012) who also reported that peer popularity was not related to their aggregate measure of preschool children’s social norm enforcement. There are several possible explanations of why this might not be the case. First, it is possible that there is no relation between children’s group-orientedness and their peer status. Yet, this explanation seems to be unlikely given that further characteristics of other-oriented behavior are positively linked to peer status (Caputi et al., 2012; Hawley and Geldhof, 2012) and given that problematic social behavior is negatively linked to peer status (Keane and Calkins, 2004).

Second, one could argue that although norm enforcement (on the long run) indeed serves the cohesion of a group and indicates the group orientedness of a species, these claims do not correctly specify the (proximal) psychological mechanisms that need to be in place for the emergence of a normative stance in early childhood. That is, children’s enforcement of social norms may not need to be based on *we-intentionality* and *group-mindedness* (Tomasello, 2014), but on other mechanisms such as social interaction experiences or domain knowledge (e.g., Piaget, 1932/1965; Turiel, 2002). Third, one could also wonder whether children’s protest in these game-like activities does actually constitute sufficient indicators for normative understanding (Brandl et al., 2015). Indeed, recent work is suggestive of a dissociation between spontaneous measure of normativity such as protest and children’s more reflected normative judgments when assessing their reflected verbal evaluation of another’s action (Wörle and Paulus, 2017). To get a more complete picture of the developmental significance of the emergence of normative understanding, it might be useful to rely on a variety of measures of early normativity.

Overall, our results add to a growing body of evidence that children’s peer status is related to different factors that include their communicative skills, their gender, and their prosocial behavior.

### What Is the Nature of Individual Differences in Children’s Social Norm Enforcement?

In addition, the current study also enabled us to examine potential correlates of children’s norm enforcement that could explain the nature of individual differences in the extent to which preschool children enforce social norms. Importantly, there was no relation to temperamental factors. Thus, our results do not provide evidence for the notion that children’s shyness explains their reluctance to protest against others’ norm violations (Rakoczy et al., 2008). However, it should be noted that the confidence interval was rather broad so that we have only an imprecise measure of the real size of the effect. In addition, we found no evidence that children’s language skills relate to their protest behavior. Finally, one could have argued that children who show more social problem behavior are less likely to enforce social norms. Again, our results do not support this notion. Our findings could suggest that there might be other factors than particular child characteristics that could explain individual differences in social norm enforcement.

How could we then explain the individual differences? Given that social norms are acquired through social interactions and shared practices (e.g., Nucci, 2004; Carpendale et al., 2013), it is possible that the nature of children’s social experiences with norms (e.g., in their family interactions) might be a key factor in explaining their inclination to enforce social norms. Indeed, first studies show that children’s experiences with peer interactions are positively related to their fair resource allocation (Paulus and Leitherer, 2017). Future research that explores children’s social environment and history of social interactions is needed to examine this possibility in greater detail.

### Limitations

The current study has also a number of limitations and leaves us with open questions. First, the current study did not assess family characteristics in greater detail. Previous research has shown that factors such as socioeconomic status and parenting styles relate to children’s social behavior (e.g., Bradley and Corwyn, 2002). Further research could examine how family characteristics relate to children’s peer status and norm enforcement. Second, it should be noted that we used teacher-report questionnaires to assess child characteristics such as temperament, pro- and antisocial behavior, and language. Although the questionnaires were developed as tools for observational assessments, partly even specifically designed for kindergarten teachers (e.g., Mayr, 2012), it should be noted that the extent to which parents and teachers are reliable observers is debated in the literature (e.g., Molina and Bulgarelli, 2012). Several aspects need to be considered. On the one hand, in contrast to parents, kindergarten teachers have usually considerable experiences with a diversity of children that allows them to more systematically compare a child’s behavior with that of his/her peers. In addition, they might be less biased by social desirability (with respect to a particular child’s behavior) than parents are. Moreover, in contrast to researchers who may observe the child in a single or at most a few visits to the kindergarten, they can aggregate their observations across a larger and more representative time scale. On the other hand, given that they are not trained observers, their observations and evaluations might suffer from the well-known biases and fallacies (see Bierhoff and Petermann, 2014). It is interesting to examine whether future research using different kind of tools would yield the same results as the current study. Third, although the current study did not reveal a significant relation between, for example, protest and peer status as well as protest and shyness, it is possible that a larger sample would have detected a smaller effect. A larger sample would also lead to narrower confidence intervals that would allow for better estimates of the real effect sizes of the relation between the measures.

## Conclusion

In sum, the current study examined the correlates of preschool children’s peer status. It demonstrates that children’s prosocial behavior and their gender were related to their peer status. No evidence was found for an impact of their norm enforcement nor was there any correlate of children’s inclination to enforce social norms.

## Ethics Statement

This study was conducted in accordance with the ethical standards laid down in the Declaration of Helsinki. Parents provided written consent before their children participated in the studies. The study followed ethical standards by the Declaration of Helsinki, but was not individually reviewed by the ethics committee as this is not obligatory at LMU Munich.

## Author Contributions

The author confirms being the sole contributor of this work and approved it for publication.

## Conflict of Interest Statement

The author declares that the research was conducted in the absence of any commercial or financial relationships that could be construed as a potential conflict of interest.

## Supplementary Material

DATA SHEET 1 Original data.
